# Supplementary material for: Intrinsic exchange biased anomalous Hall effect in an uncompensated antiferromagnet MnBi2Te4
Source: Nat Commun. 2024 Apr 3;15:2881. doi: 10.1038/s41467-024-46689-8 (PMC10991375; doi:10.1038/s41467-024-46689-8)
Supplement: Supplementary file 1 — Supplementary Information [file 41467_2024_46689_MOESM1_ESM.pdf]

Supplementary Information

Intrinsic exchange biased anomalous Hall effect in an uncompensated antiferromagnet $MnBi_2Te_4$

Su Kong Chong[1†*], Yang Cheng[1†], Huiyuan Man[2,3], Seng Huat Lee[6,7], Yu Wang[6,7], Bingqian Dai[1], Masaki Tanabe[1], Ting-Hsun Yang[1], Zhiqiang Mao[6,7], Kathryn A. Moler[2,4,5] and Kang L. Wang[1]*

[1]Department of Electrical and Computer Engineering, University of California, Los Angeles, California 90095, United States

[2]Geballe Laboratory for Advanced Materials, Stanford University, Stanford, California 94305, USA

[3]Stanford Nano Shared Facilities, Stanford University, Stanford, CA 94305, USA

[4]Stanford Institute for Materials and Energy Sciences, SLAC National Accelerator Laboratory, Menlo Park, California 94025, USA

[5]Department of Physics and Applied Physics, Stanford University, Stanford, California 94305, USA

[6]2D Crystal Consortium, Materials Research Institute, The Pennsylvania State University, University Park, PA 16802, USA

[7]Department of Physics, The Pennsylvania State University, University Park, PA 16802, USA

*Corresponding authors: sukongc@g.ucla.edu; wang@seas.ucla.edu

[†]These authors contributed equally to this work

I. Device details

Table S1. List of the details for the 7SL MBT devices fabricated in the study.

Label	Fabrication method	Effective area (±5%)	Max. exchange bias
D1	Top contact	150 μm^2	99 mT (FC)
D2	Bottom contact	80 μm^2	6.2 mT (FT)
D3	Top contact + etching	60 μm^2	339 mT (FT)
D4	Top contact	170 μm^2	360 mT (FT)
D5	Top contact + etching	40 μm^2	406 mT (FT);<br>45 mT (FC)
D6	Top contact + etching	70 μm^2	0.5 mT (FT)

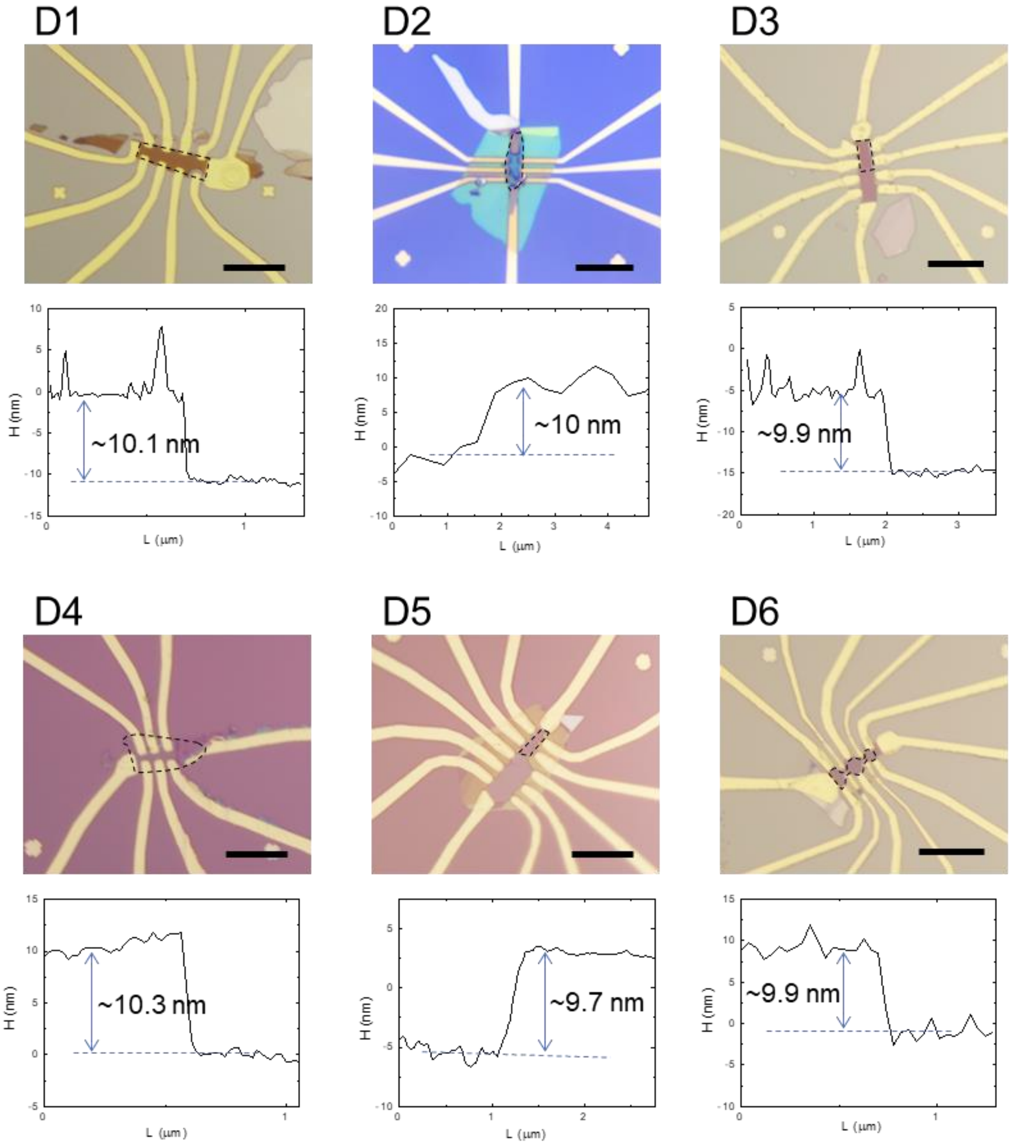

Figure S1. Optical images (top) and height profiles (bottom) measured by atomic force microscopy for the different 7SL MBT devices (D1-D6). The scale bar in the optical images is 20 μm. All the devices show a thickness of ~10 nm, corresponding to the 7SL MBT (1SL~ 1.4 nm).

II. Magnetic phase transitions

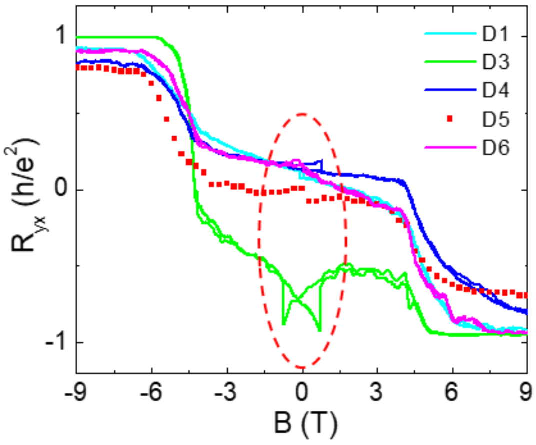

Figure S2. R_{yx} as a function of magnetic field for the different MBT devices (D1, D3-D6) measured at 2K at their gate voltages controlled near their charge neutrality regions. The quality of the studied devices is proven by the nearly quantized Chern insulator state in the spin-alignment phase at a high magnetic field >7T with R_{yx} exceeding 80% of the quantization value (in h/e^2) at 2K.

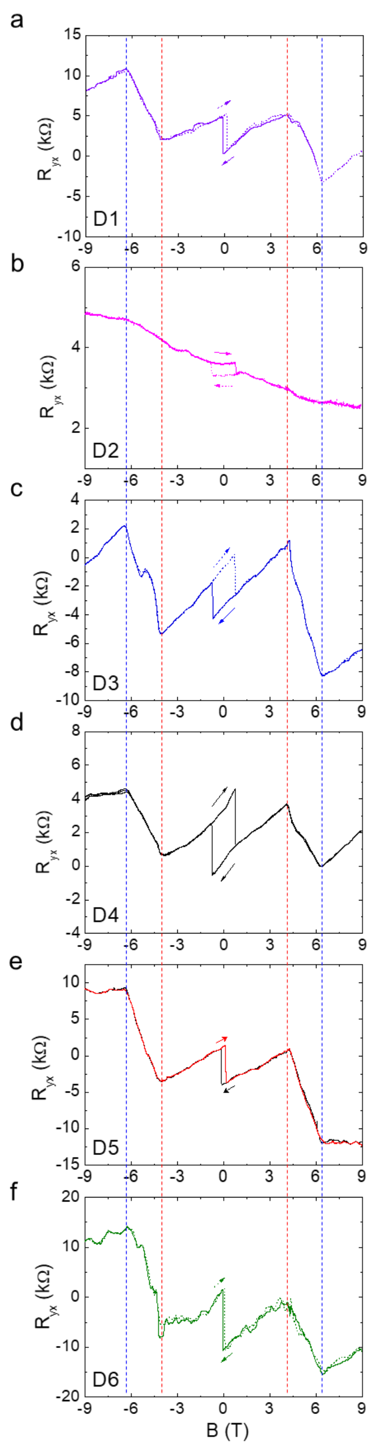

Figure S3. Magnetic field dependent R_{yx} taken at a full field sweep for the different MBT devices (a) D1, (b) D2, (c) D3, (d) D4, (e) D5 and (f) D6 measured at 2K at the gate voltages controlled to the hole conduction regime, except for D2 due to gate leakage. The red and blue dashed lines trace the magnetic transition fields for spin-flop (H_1) and spin-flip (H_2), respectively, as determined from the change in slope of the magnetic field-dependent R_{yx} curves.

III. Gate voltage, temperature and cooling field dependence

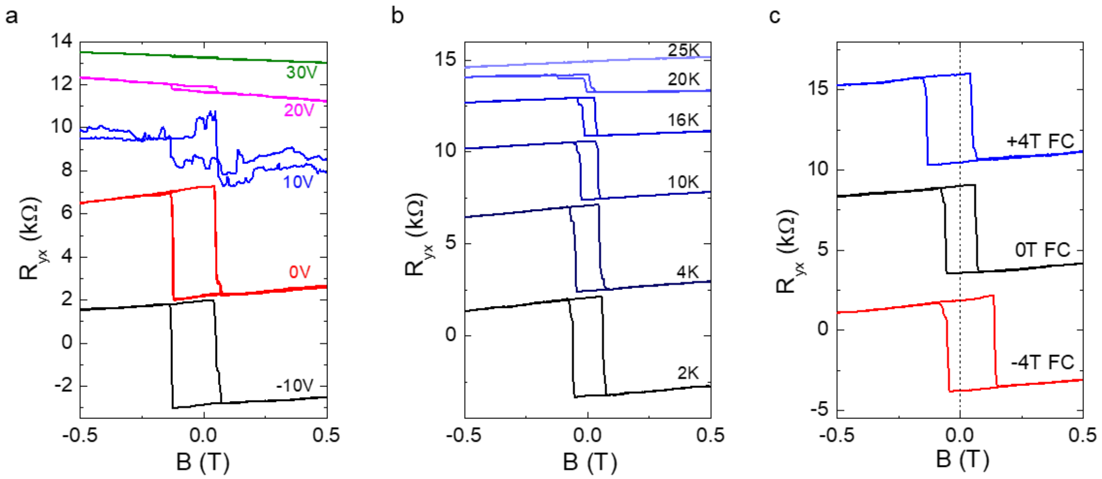

Figure S4. Plots of the R_{yx} hysteresis loops for the MBT device D5 measured at different (a) gate voltages, (b) temperatures, and (c) cooling fields. The gate dependent R_{yx} hysteresis loops in (a) show clearly the transition from hole to electron conductions as the gate voltage increases from -10V to +30V, as indicated by the change from positive to negative slopes at the saturation of R_{yx} curves. The coercivity is nearly independent of the gate voltage. The temperature dependent R_{yx} hysteresis loops in (b) show the fully vanish R_{yx} hysteresis loop at a temperature above the Neel transition, indicating that the anomalous Hall effect is mainly contributed by the uncompensated layer in the AFM. The field cooling results in a negative exchange bias together with the enhancement in H_C, consistent with the observation of the field-cooled exchange bias in device D1.

IV. Field training for an even SL MBT device

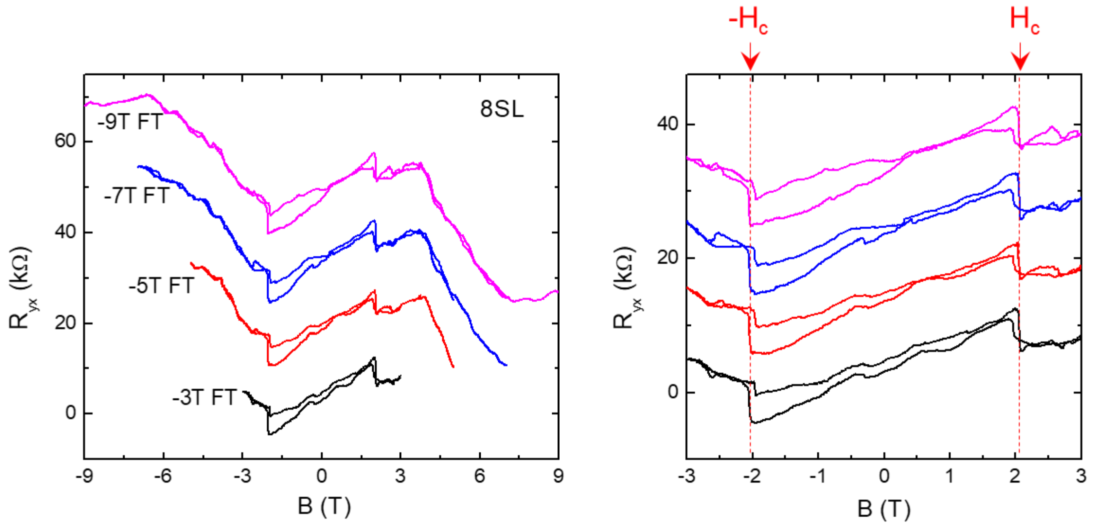

Figure S5. Field training effect for an 8SL MBT device. Magnetic field dependent R_{yx} taken at different training fields for the 8SL MBT device. The red dashed lines trace the magnetic spin-flop transition field (H_1). The R_{yx} hysteresis loops remain unchanged over the different training fields, which negates the field training effect in the compensated AFM of the 8SL MBT.

V. Effect of field initialization in field training

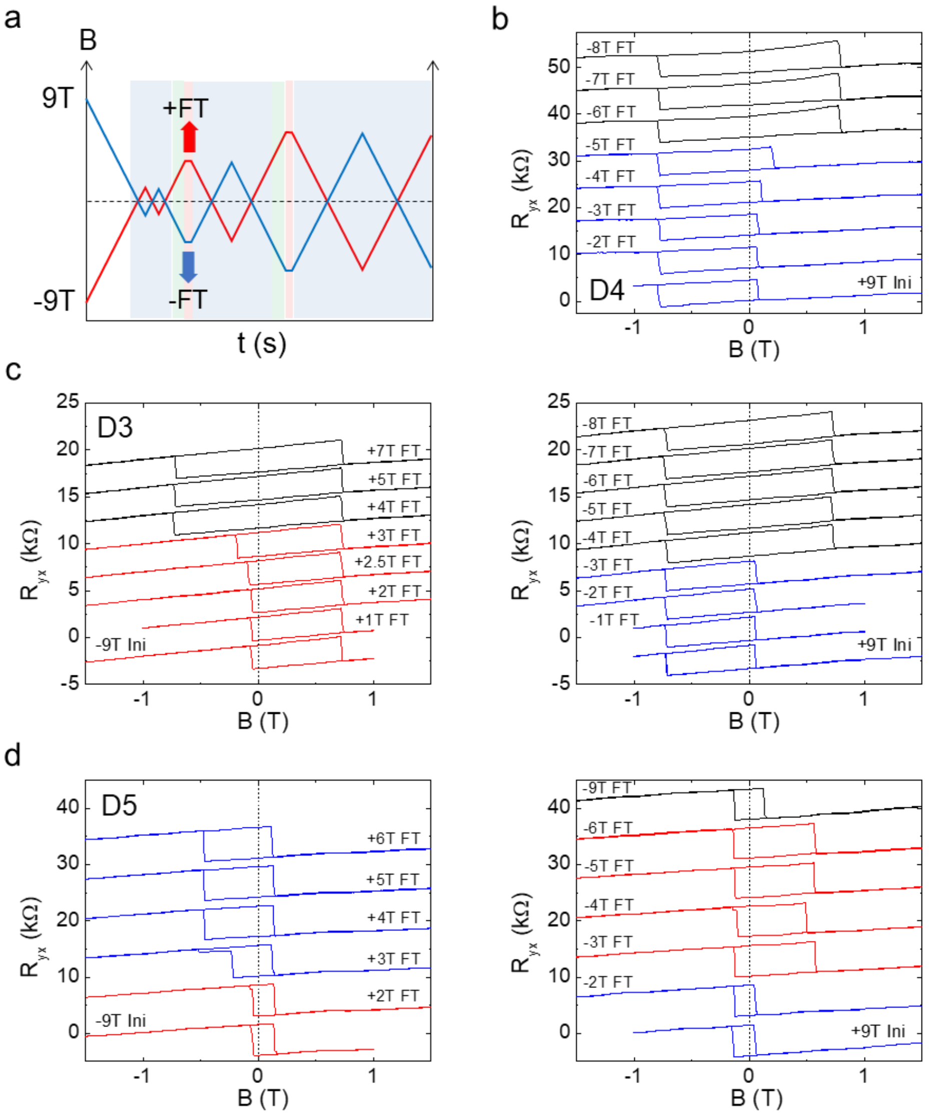

Figure S6. (**a**) Illustration of the field training protocol for the setting of temperature and magnetic field as a function of time. The red and blue curves represent the field sweep sequence for positive and negative field training, respectively. The green, red and blue shades denote the field set, field train and field sweep regions, respectively. Plots of R_{yx} hysteresis loops in different magnetic field strengths under (Left) -9T, and (Right) +9T initialization, followed by the field training protocols for MBT devices of (**b**) D4, (**c**) D3, and (**d**) D5. All data were taken at 2K. The R_{yx} versus magnetic field curves in all panels is shifted vertically for comparison. The training fields for each curve are labeled in the figures. The R_{yx} hysteresis loops with positive and negative exchange bias fields (H_{EB}) are plotted in red and blue curves, respectively.

VI. Charge carrier independent field training

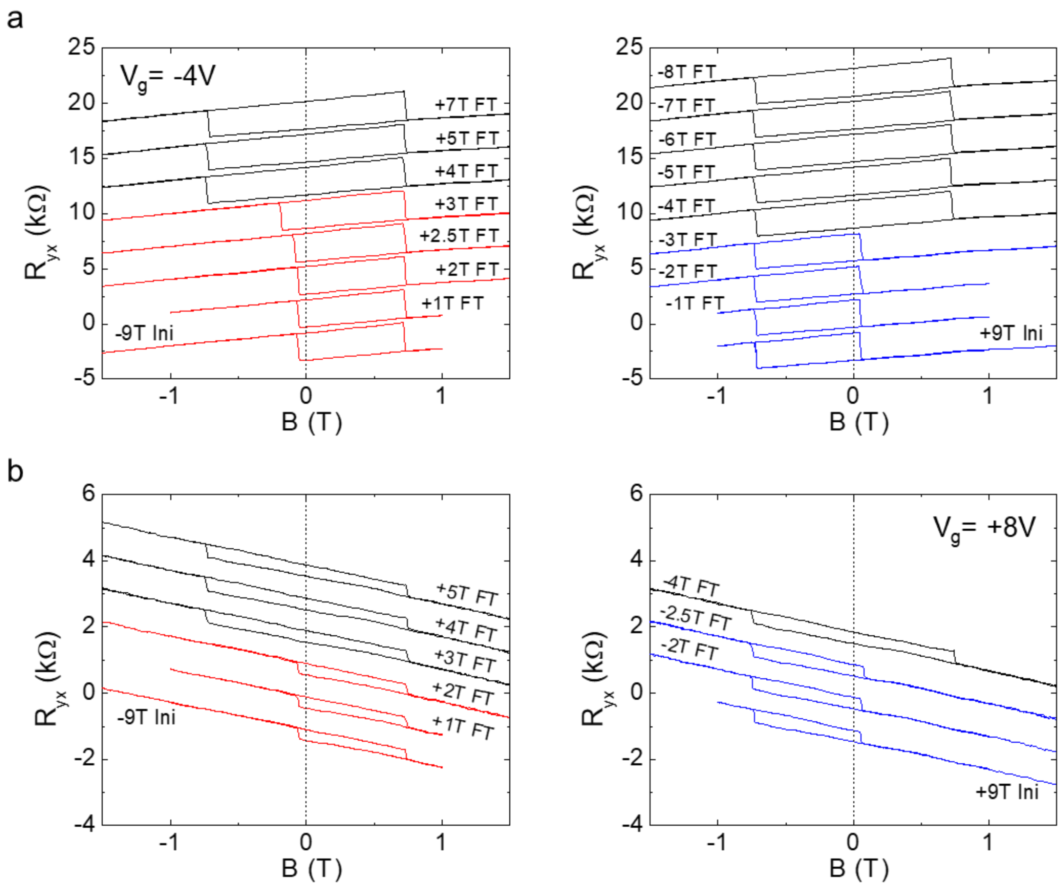

Figure S7. Plots of R_{yx} hysteresis loops in different magnetic field strengths under (Left) -9T, and (Right) +9T initialization, followed by the field training protocols for the MBT device D3 measured at different gate voltages (V_g) of (**a**) -4V, and (**b**) +8V. The positive and negative slopes of R_{yx} with magnetic field are resulting from the hole and electron carriers, respectively, as controlled by gate voltages. The coercive field (H_C) and exchange bias fields (H_{EB}) are nearly carriers independent.

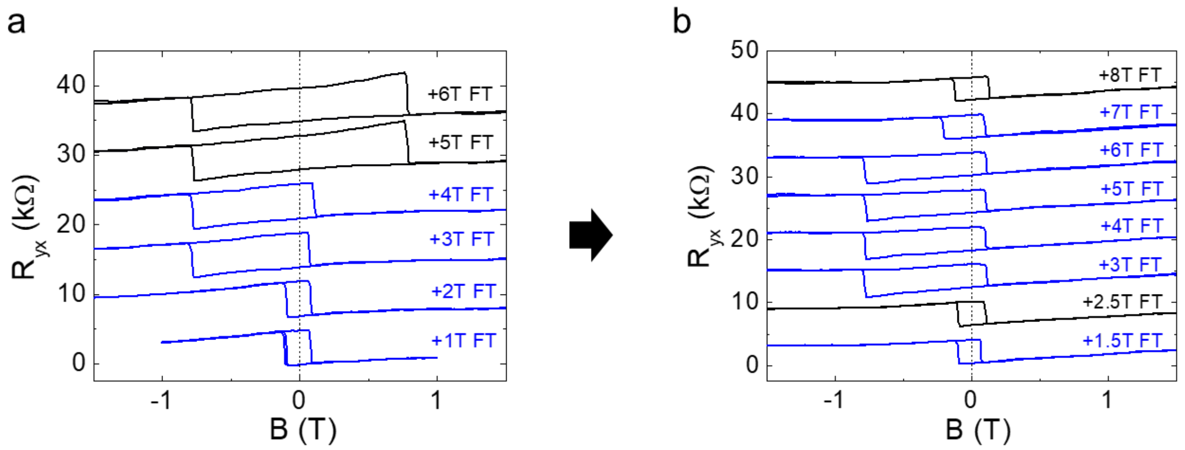

Figure S8. Plots of R_{yx} hysteresis loops in the magnetic field under zero field initialization at positive ($+H_{FT}$) field training protocols for the device D4 measured in the (**a**) first cooling (as-prepared), and (**b**) second cooling (about one month after the first cooling, sample kept in controlled Ar environment with O_2 ~1 ppm). All data were taken at temperature of 2K. The R_{yx} versus magnetic field curves in all panels is shifted vertically for comparison. The training fields for each curve are labeled in the figures.
